# Lymphomatoid Granulomatosis with Paraneoplastic Polymyositis: A Rare Malignancy with Rare Complication

**DOI:** 10.1155/2016/8242597

**Published:** 2016-02-04

**Authors:** Shanley O'Brien, Paul Schmidt

**Affiliations:** ^1^Department of Internal Medicine, The University of Kansas Medical Center, 3901 Rainbow Boulevard, Kansas City, KS 66160, USA; ^2^Department of Internal Medicine, Division of Allergy, Clinical Immunology & Rheumatology, The University of Kansas Medical Center, 3901 Rainbow Boulevard, Kansas City, KS 66160, USA

## Abstract

Lymphomatoid granulomatosis is a rare Epstein-Barr virus driven lymphoproliferative disease. It most commonly presents with symptoms of lung involvement such as cough, chest tightness, and dyspnea or constitutional symptoms of weight loss, malaise, and fever. The diagnosis is obtained by biopsy and histopathology. Here we report the case of a 31-year-old male who presented with weight loss, rash, and weakness and was diagnosed with lymphomatoid granulomatosis with paraneoplastic polymyositis. We explore the relationship of Epstein-Barr virus with inflammatory myopathy and discuss paraneoplastic inflammatory myopathy.

## 1. Introduction

Lymphomatoid granulomatous is a rare Epstein-Barr virus (EBV) driven lymphoproliferative disease first described in 1972 [1]. Because it is an angiocentric and angiodestructive process, it was initially described as a cross between angiitis and lymphoma. It results in atypical lymphoid cell accumulation within tissues, which presents as infiltrative nodular lesions [2]. It most commonly affects patients from ages 30 to 50. It affects males more than females [1]. Cough, chest pain, chest tightness, and dyspnea are common presenting symptoms since the lungs are involved in >90% of cases at the time of diagnosis [2]. Alternatively, patients can present with constitutional symptoms such as weight loss, malaise, and fever [2]. Lymphomatoid granulomatosis is a multisystem disease that affects the lungs, skin, CNS, kidney, liver, spleen, and lymphatics [2]. Tissue biopsy of affected organs is required to accurately diagnose lymphomatoid granulomatosis [2, 3].

Here we report the case of a man who was presented with rash, weakness, and weight loss. He was diagnosed with lymphomatoid granulomatosis and paraneoplastic polymyositis. While there is a case of lymphomatoid granulomatosis with primary invasion of the skeletal muscles documented in the literature, our case is the first documented of paraneoplastic polymyositis with this very rare malignancy [4].

## 2. Case Report

A 31-year-old Hispanic male presented to his family doctor with a 6-month history of a painful, nonpruritic nodular rash with ulcerations and necrosis on his forearms, shins, and thighs. Concurrently over the previous 4 months he had developed weakness, making it difficult for him to raise his arms, go upstairs, and get up from a seated position. He also had unintentional weight loss, fatigue, low-grade fever, and a swelling in his right groin. Skin biopsy showed an atypical T cell infiltrate forming expansive nodules in the adventitial dermis favoring a diagnosis of T cell lymphoma. CT revealed diffuse axillary, subcutaneous, pulmonary, hepatic, renal, and splenic nodules and masses (Figure 2). He was admitted to the hospital for further workup.

**Figure 1:**
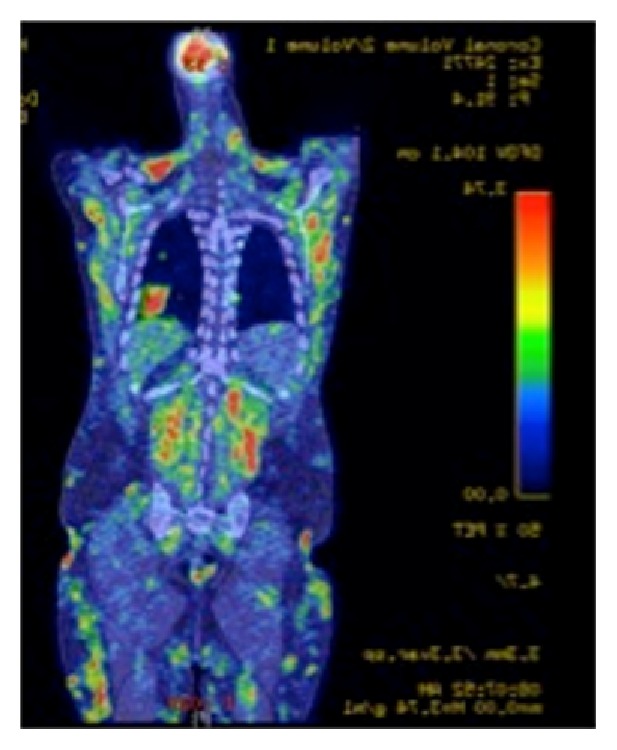
PET scan. PET scan with innumerable intramuscular and subcutaneous foci of elevated uptake throughout the neck, chest, abdomen, pelvis, proximal upper extremities, and proximal lower extremities.

**Figure 2:**
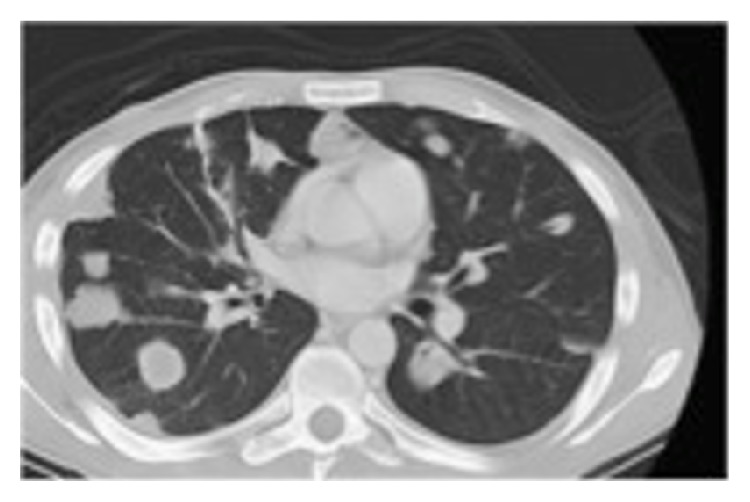
CT Chest. CT chest with diffuse pulmonary nodules.

On arrival to the hospital, he had significant proximal muscle weakness and numerous violaceous plagues and nodules on his forearms, abdomen, and legs, two of which had a necrotic center. Distal muscle strength was normal. He had several abnormal elevations in his laboratory results, including CK of 696 U/L (nl 35–232 U/L), Aldolase of 44 U/L (nl <7.7 U/L), erythrocyte sedimentation rate (ESR) of 17 MM/Hr (nl 0–15 MM∖Hr), C-reactive protein of 5.75 MG/DL (nl <1.0 MG/DL), and lactate dehydrogenase (LDH) of 365 U/L (nl 100–210 U/L). The patient also had anemia and leukopenia. EBV PCR was positive with 7,200 blood copies/mL. Autoimmune workup including ANA and myositis antibody panel was negative. EMG of the right transverse abdominis and vastus lateralis muscles showed evidence of mild irritative myopathy. Right peroneal and tibial motor studies showed decreased amplitude due to the severe edema in the legs. Right median, ulnar, and sural studies were normal. PET scan showed innumerable intramuscular and subcutaneous foci of elevated uptake throughout the visualized neck, chest, abdomen, pelvis, proximal upper extremities, and proximal lower extremities (Figure 1).

**Figure 3:**
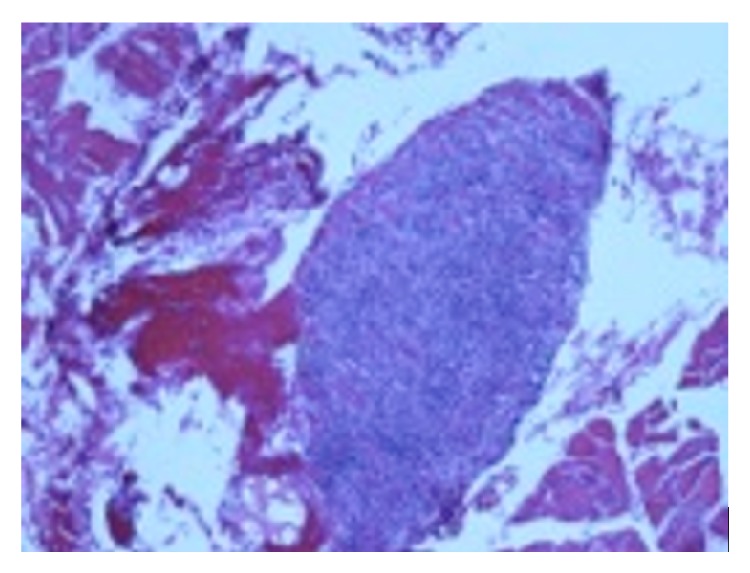
Muscle biopsy. Biopsy of the left vastus lateralis muscle with marked endomysial infiltrates of predominantly small well-differentiated lymphocytic inflammatory cells. The skeletal muscle is overrun by the lymphocytic infiltrates. Multiple aggregates of these lymphocytic infiltrates are present.

He then had a liver biopsy that showed atypical lymphoid proliferation consistent with lymphomatoid granulomatosis, grade 2. A left lung wedge resection found lymphomatoid granulomatosis grade 3 with features suggestive of plasmablastic transformation. Both the liver and lung biopsies were positive for EBV. Muscle biopsy of the left vastus lateralis muscle found marked lymphocytic endomysial infiltrates consistent with severe active inflammatory myopathy (Figure 3). Muscle biopsy was negative for EBV.

The patient was diagnosed with lymphomatoid granulomatosis, grade 3, stage IV with B symptoms and paraneoplastic polymyositis. He was started on rituximab and prednisone in the hospital. His initial treatment plan included R-CHOP (rituximab plus cyclophosphamide, doxorubicin, vincristine, and prednisone) for 4 cycles with the goal of going to allogenic transplant during the first complete remission. His response to R-CHOP was suboptimal on PET after his second cycle and he switched to R-HyperCVAD (rituximab plus hyperfractionated cyclophosphamide, vincristine, doxorubicin, and dexamethasone alternating with high-dose methotrexate and cytarabine). After 1 cycle of R-HyperCVAD, he proceeded to matched sibling donor stem cell transplant. Bone marrow biopsy 6 months after transplant showed no evidence of lymphoma. His proximal muscle weakness was significantly improved at that time as well.

## 3. Discussion

Lymphomatoid granulomatosis is commonly diagnosed in patients with immunodeficiency, such as those with Wiskott-Aldrich, HIV infection, or allogenic organ transplant [2, 3]. Impaired host cellular immunity is responsible for the proliferation of EBV infected B cells in patients with lymphomatoid granulomatosis. Without proper cellular immunity, the EBV infected B cells are allowed to transition from being latent to being malignant [3]. While the role of EBV in lymphomatoid granulomatosis is well established, there is only a theoretical association of EBV with inflammatory myopathy.

One hypothesis is that EBV plays a role in the pathogenesis of inflammatory myopathy in patients both with and without malignancy [5]. Studies have demonstrated higher levels of EBV antibodies and EBV DNA in patients with polymyositis and dermatomyositis [5, 6]. Levels were higher still in those patients with inflammatory myopathy who also had malignancy [5]. While these results suggest a correlation of EBV with inflammatory myopathy, they did not clearly establish a causative relationship. In the patients with malignancy, it was unclear whether the inflammatory myopathy occurred as a paraneoplastic process or was simply coincidental [5].

Inflammatory myopathy has been documented in patients with chronic active EBV. In these cases, muscle inflammation is caused by direct invasion of the EBV positive cells. Tsutsumi et al. report a case of biopsy proven muscular invasion by EBV positive T cells in a patient with chronic active EBV. The patient described had no noted weakness and did not improve with steroid treatment [7]. Shirasaki et al. reported another case of chronic active EBV with muscle invasion on biopsy. After initial improvement with prednisolone, the patient had an aggressive relapse 3 years later [8]. Furthermore, Nakamura reported 3 additional cases of patients with chronic generalized myositis associated with chronic active EBV. In all three, oligoclonal expansion of EBV infected T cell lymphocytes was seen in the muscle biopsy without evidence of malignancy. Akin to other reports of myositis associated with chronic active EBV, these three patients also had poor response to corticosteroids and other immunosuppressants [9].

Cancer associated rheumatic diseases were first described in 1916 [10]. The most frequently reported paraneoplastic rheumatic diseases are inflammatory myopathies, seronegative rheumatoid arthritis, and some atypical vasculitides [10]. The highest incidence of malignancy in those with inflammatory myopathy is in the 2 years both before and after the diagnosis [11]. Cancer associated polymyositis is most strongly associated with non-Hodgkin lymphoma and lung and bladder cancer [11, 12]. Cases of paraneoplastic inflammatory myopathy have also been observed in several other solid tumors [10]. However, we are not aware of any cases of inflammatory myopathy in lymphomatoid granulomatosis. It is very important to be aware of the association between rheumatic syndromes and malignancy as they are sometimes the first sign of cancer. Age appropriate cancer screening along with a detailed history and physical exam should take place to avoid missing a malignancy [11]. Furthermore, an undiagnosed malignancy should be reconsidered in patients with resistant disease. The treatment of paraneoplastic rheumatic disease is treatment of the underlying malignancy [10, 11].

## 4. Conclusion

This is the first known case reported of lymphomatoid granulomatosis with paraneoplastic polymyositis. This case illustrates the need to better understand what role EBV has in inflammatory myopathy. Additionally, it demonstrates the importance of searching for malignancy in patients with an atypical presentation of rheumatic disease. In cases of paraneoplastic rheumatic disease, improvement is expected only after treatment of the underlying malignancy.

## References

[1] Raja R., Lamont D., Yung A., Solanki K. (2010). A can of red herrings. *International Journal of Rheumatic Diseases*.

[2] Roschewski M., Wilson W. H. (2012). Lymphomatoid granulomatosis. *Cancer Journal*.

[3] Dunleavy K., Roschewski M., Wilson W. H. (2012). Lymphomatoid granulomatosis and other Epstein-Barr virus associated lymphoproliferative processes. *Current Hematologic Malignancy Reports*.

[4] Schmalzl F., Gasser R. W., Weiser G., Zur Nedden D. (1982). Lymphomatoid granulomatosis with primary manifestation in the skeletal muscular system. *Klinische Wochenschrift*.

[5] Chen D.-Y., Chen Y.-M., Lan J.-L. (2010). Polymyositis/dermatomyositis and nasopharyngeal carcinoma: the Epstein-Barr virus connection?. *Journal of Clinical Virology*.

[6] Barzilai O., Sherer Y., Ram M., Izhaky D., Anaya J. M., Shoenfeld Y. (2007). Epstein-Barr virus and cytomegalovirus in autoimmune diseases: are they truly notorious? A preliminary report. *Annals of the New York Academy of Sciences*.

[7] Tsutsumi S., Ohga S., Nomura A. (2002). CD4^−^CD8^−^ T-cell polymyositis in a patient with chronic active Epstein-Barr virus infection. *American Journal of Hematology*.

[8] Shirasaki F., Taniuchi K., Matsushita T., Hamaguhi Y., Takata M., Takehara K. (2002). Epstein-Barr virus-associated T-cell lymphoma: a case of eyelid swelling and intramuscular infiltration mimicking dermatomyositis. *British Journal of Dermatology*.

[9] Uchiyama T., Arai K., Yamamoto-Tabata T. (2005). Generalized myositis mimicking polymyositis associated with chronic active Epstein-Barr virus infection. *Journal of Neurology*.

[10] Racanelli V., Prete M., Minoia C., Favoino E., Perosa F. (2008). Rheumatic disorders as paraneoplastic syndromes. *Autoimmunity Reviews*.

[11] Azar L., Khasnis A. (2013). Paraneoplastic rheumatologic syndromes. *Current Opinion in Rheumatology*.

[12] Tsunemine H., Maruoka H., Akasaka H. (2013). Polymyositis as a paraneoplastic syndrome in cytotoxic molecule-positive and Epstein-Barr virus-associated peripheral T-cell lymphoma, not otherwise specified. *Internal Medicine*.

